# The decision tool unipolar depression (DTUD): a new measure to facilitate the early identification of patients with major depressive disorder in need of highly specialized care

**DOI:** 10.1186/s12888-019-2165-9

**Published:** 2019-06-11

**Authors:** Frédérique C. W. van Krugten, Maartje Goorden, Anton J. L. M. van Balkom, Patricia van Oppen, Henricus G. Ruhé, Digna J. F. van Schaik, Werner B. F. Brouwer, Leona Hakkaart-van Roijen, on behalf of the Decision Tool Unipolar Depression Consortium, A. J. L. M. van Balkom, C. L. H. Bockting, T. M. van den Boogaard, D. J. F. van Schaik, P. van Oppen, H. G. Ruhé, F. P. M. L. Peeters, J. Spijker, P. A. J. M. Huijs, M. A. J. M. Loo, H. L. Van, W. J. Broekema, A. E. van’t Thijs-van’t Hoog

**Affiliations:** 1 Erasmus School of Health Policy & Management, Erasmus University Rotterdam, PO Box 1738, 3000 DR Rotterdam, The Netherlands; 2 Department of Psychiatry, Amsterdam UMC, Vrije Universiteit Amsterdam, Amsterdam, The Netherlands; 3 Amsterdam Public Health Research Institute, Amsterdam, The Netherlands; 4 Academic Outpatient Clinic, GGZ InGeest, Amsterdam, The Netherlands; 5 Department of Psychiatry, Academic Medical Center, Amsterdam, The Netherlands; 6 Department of Psychiatry, Radboud University Medical Center, Nijmegen, The Netherlands

**Keywords:** Major depressive disorder, Depression, Early intervention, Treatment stratification, Measurement, Psychometrics, Validation, Tertiary healthcare

## Abstract

**Background:**

Selection of the optimal initial treatment in patients with major depressive disorder (MDD) in need of highly specialized care has the potential to benefit treatment outcomes and cost-effectiveness of treatment strategies. However, to date, there is a paucity of measures that could guide the selection of the initial treatment, in particular to indicate which patients with MDD are in need of highly specialized care. Recognizing this gap, this paper reports on the development and psychometric evaluation of the Decision Tool Unipolar Depression (DTUD), aimed to facilitate the early identification of patients with MDD in need of highly specialized care.

**Methods:**

The DTUD was developed using a mixed-methods approach, consisting of a systematic review and a concept mapping study. To evaluate the psychometric features of the DTUD, a cross-sectional multicenter study was conducted. A total of 243 patients with MDD were evaluated with the DTUD. Feasibility was operationalized as the time required to complete the DTUD and the content clarity of the DTUD. Inter-rater reliability was evaluated using Krippendorf’s alpha. The Maudsley Staging Method (MSM) and the Dutch Measure for quantification of Treatment Resistance in Depression (DM-TRD) were administered to assess the convergent validity. A receiver operator characteristic curve was generated to evaluate the criterion validity and establish the optimal cut-off value.

**Results:**

The mean administration time was 4.49 min (SD = 2.71), and the content of the total DTUD was judged as clear in 94.7% of the evaluations. Inter-rater reliability values ranged from 0.69 to 0.91. Higher scores on the DTUD were associated with higher scores on the MSM (r_s_ = 0.47) and DM-TRD (r_s_ = 0.53). Based on the maximum Youden index (0.494), maximum discrimination was reached at a cut-off score of ≥5 (sensitivity 67%, specificity 83%).

**Conclusion:**

The DTUD demonstrated to be a tool with solid psychometric properties and, therefore, is a promising measure for the early identification of patients with MDD in need of highly specialized care. Use of the DTUD has the potential to facilitate the selection and initiation of the optimal initial treatment in patients with MDD, which in turn may improve the clinical effectiveness and cost-effectiveness of treatment strategies.

**Electronic supplementary material:**

The online version of this article (10.1186/s12888-019-2165-9) contains supplementary material, which is available to authorized users.

## Background

Since delayed initiation of appropriate treatment in patients with major depressive disorder (MDD) has been associated with relapse, recurrence and chronicity [1–3], early initiation of the optimal type and intensity of intervention is considered essential [4]. The stepped care model of healthcare delivery, according to which many parts of healthcare systems are organized and sometimes incentivized to work [5], may however delay the initiation of the optimal type and intensity of intervention. Within the stepped care approach, patients first receive the briefest, least intrusive, or least costly intervention, and only ‘step up’ the treatment pathway in case of changing treatment needs or insufficient health gains from initial treatment [6]. Although the stepped care model of healthcare delivery is considered an appropriate approach in patients who recover with low intensity treatments [7, 8], the effectiveness and cost-effectiveness of the stepped care model is questionable in patients who, identifiably, are in need of high intensity treatment [6]. Subsequent referral of these patients to highly specialized mental healthcare (i.e. tertiary mental healthcare) is likely to prolong the treatment course and compromise clinical and functional outcomes and cost-effectiveness of treatments. Selection of the optimal initial treatment in patients with MDD in need of highly specialized care is therefore warranted, as it can improve the effectiveness and cost-effectiveness of treatment paths, but strongly relies on the availability of psychometrically sound instruments to aid clinicians in the early identification of these patients [4, 9].

Several measures are available to screen for MDD and assess its severity in clinical and research settings [10–12]. However, to date, there is a paucity of measures that facilitate the selection of the optimal initial treatment, in particular to indicate which patients with MDD are in need of highly specialized care. Recognizing this gap, in this paper we report on the development and psychometric evaluation of the Decision support Tool for the assessment of highly specialized mental healthcare needs of patients with a Unipolar Depression, or the “Decision Tool Unipolar Depression” (DTUD) for short. The DTUD is a 10-item clinician-administered instrument to facilitate the early identification of patients with MDD in need of highly specialized care. The focus of this paper is on describing the development of the DTUD and presenting the first results regarding its feasibility, inter-rater reliability, convergent validity, and criterion validity.

## Methods

### Definition of terms

As illustrated by the WHO’s Optimal Mix of Services Pyramid [13], most people with mental health problems are ideally treated in primary care services. When the mental health needs require intervention beyond that which can be provided by primary care services, the patient should be referred to specialized mental healthcare services (i.e. secondary mental healthcare) [13]. Specialized mental healthcare includes the mental health services provided in community mental health centers and general hospitals [13]. Highly specialized mental healthcare (i.e. tertiary mental healthcare) includes specialized interventions provided by highly-trained mental healthcare professionals with expertise in a given area to patients with mental health problems that require intervention over and above those provided in specialized mental healthcare [14, 15]. Given the required level of staff expertise, management, security, and resources of highly specialized mental healthcare, those services are frequently, but not necessarily, affiliated with academic medical healthcare centers [14].

### Decision tool unipolar depression (DTUD) development

Aim of the development of the DTUD was to create a valid and reliable, yet at the same time short and easy to score clinician-administered measure to facilitate the early identification of the subgroup of patients with MDD who are in need of highly specialized mental healthcare. The development of the DTUD comprised the following three phases: (i) identification of indicators of patients with MDD in need of highly specialized care through a systematic literature review, (ii) development of a conceptual framework to inform item generation, and (iii) development of the measure and evaluation of face validity and feasibility.

In the first phase of the development of the DTUD, a systematic literature review was carried out to provide a scientific foundation for the selection of items included in the resultant measure [16]. The PubMed and PsycINFO electronic databases were searched for studies published between January 2000 and January 2015 reporting indicators of patients with MDD in need of highly specialized care. The search retrieved 7360 references, of which 16 met the inclusion criteria. Two reviewers determined study eligibility, reviewed study quality, and performed data abstraction. From the included studies, 48 indicators of patients with MDD in need of highly specialized care were abstracted. For more details on the systematic review we refer to Van Krugten et al. [16].

In the second phase of the development of the DTUD, concept mapping methodology [17] was used to generate a conceptual framework to guide tool development [18]. In total, 67 MDD experts participated in the subsequent steps of the concept mapping process. During the first step of the concept mapping process (i.e. the brainstorming step), participating experts were asked to review the indicators from the systematic literature review, and, when necessary, add additional indicators that could discriminate MDD patients with and without a highly specialized care need. In the second step of the concept mapping process (i.e. the sorting step), participants individually sorted the resulting indicators from the brainstorming step into conceptual groupings. The data from the sorting step were analyzed using nonmetric multidimensional scaling and agglomerative hierarchical cluster analyses, resulting in a ten-cluster concept map solution. In a consensus meeting, consortium members reviewed the concept map and assigned labels to each of the ten clusters. The ten clusters (i.e. overarching domains) of indicators of patients with MDD in need of highly specialized care were assigned the following labels: depression severity, onset and (treatment) course, comorbid personality disorder, comorbid substance use disorder, other psychiatric comorbidity, somatic comorbidity, maladaptive coping, childhood trauma, social factors, and psychosocial dysfunction. For more details on the concept mapping study we refer to Van Krugten et al. [18].

In the third phase of the development of the DTUD, members of Decision Tool Unipolar Depression Consortium generated the draft DTUD based on the resulting overarching domains from the concept mapping study (phase ii). In a consensus meeting, each of the overarching domains was operationalized as a dichotomous item. In order to evaluate the feasibility and face validity of the DTUD, the draft version of the DTUD was pilot-tested in a convenience sample of 46 patients aged 18 years or older with a (principal) primary diagnosis of MDD referred for treatment to a specialized or highly specialized treatment center in the Netherlands. Participating clinicians were asked to complete a web-based survey comprising the draft version of the DTUD, comment on the clarity of content of the DTUD and register the time needed to complete the DTUD. In a 3-h consensus meeting, consortium members reviewed the pilot data and made minor revisions to the wording of the draft version, resulting in the final version of the DTUD. The resulting DTUD is a 10-item clinician-administered instrument designed to facilitate the early identification of individual patients with MDD in need of highly specialized mental healthcare. Each item has two response options (“Yes” and “No”). The total score is calculated by summing the scores of the ten items and ranges from 0 to 10. The abbreviated items of the DTUD are listed in Table 1. An English translation of the complete DTUD is presented in Additional file 1.

**Table 1:** Items, response options and scoring system of the DTUD

Item^a^		Response options	Score
1	Severe depression	Yes	1
		No	0
2	Previous unsuccessful treatment of the index depressive episode in specialized care *and* a recurrent *or* chronic course	Yes	1
		No	0
3	Treatment-interfering comorbid personality disorder	Yes	1
		No	0
4	Treatment-interfering comorbid substance use disorder	Yes	1
		No	0
5	Other treatment-interfering psychiatric comorbidity	Yes	1
		No	0
6	Treatment-interfering somatic comorbidity	Yes	1
		No	0
7	Treatment-interfering maladaptive coping	Yes	1
		No	0
8	Severe or longstanding childhood trauma	Yes	1
		No	0
9	Social factors maintaining the depression	Yes	1
		No	0
10	Severe psychosocial dysfunctioning	Yes	1
		No	0

^a^ Item text is abbreviated. An English translation of the complete DTUD is presented in Additional file 1

### Study design and population

The aim of the present study was to evaluate the psychometric properties of the DTUD. To that end, a cross-sectional, observational multicenter study was carried out in six psychiatric specialized and highly specialized outpatient centers in The Netherlands. The Medical Ethical Committee of the Erasmus University Medical Center Rotterdam reviewed and approved the study (MEC-2015-670).

243 randomly selected outpatients referred for treatment of a current episode of MDD to one of the six participating sites were evaluated with the DTUD under routine care conditions. Study inclusion criteria were: aged 18 years or older and a primary (principal) diagnosis of MDD according to Diagnostic and Statistical Manual of Mental Disorders (DSM)-IV criteria. The DSM-IV axis I diagnosis was determined by the administration of a Dutch version of the Structured Clinical Interview for DSM-IV Axis I Disorders (SCID-I) [19] or by a structured clinical interview using DSM-IV criteria.

### Measures

In addition to the DTUD, the following instruments were administered:

The *Maudsley Staging Method (MSM)* [20] is a five-item, clinician-administered instrument designed to quantify (future) treatment resistant depression (TRD). The MSM comprises the following three dimensions: duration, severity and failed treatments in current episode of depression. The total score ranges from 3 to 15, and may be categorized into three staging categories: mild (3–6), moderate (7–10), and severe (11–15).

The *Dutch Measure for quantification of Treatment Resistance in Depression (DM-TRD)* [21] is an eleven-item, clinician-administered instrument, and an extension of the MSM. In addition to the MSM dimensions, the DM-TRD comprises dimensions for functional impairment, comorbid anxiety and personality disorders and psychosocial stressors. The total score ranges from 2 to 27, with higher values indicating higher levels of TRD.

### Procedures

Patients who were referred to one of the six participating clinics with a primary (principal) diagnosis of MDD were evaluated with the DTUD. Attending clinicians completed the DTUD at the end of the diagnostic phase, on the basis of the diagnostic results. In addition to the DTUD, the clinician administered the MSM and DM-TRD, recorded the patients’ basic demographic information (age, sex), and answered two questions regarding the feasibility of the DTUD. The participating clinics entered the data in completely anonymized web-based case report forms as approved by the institutional review board.

Feasibility was operationalized as the time required to complete the DTUD, and the content clarity of the DTUD. Completion time was considered acceptable if the mean time taken to complete the DTUD was ≤10 min. The clarity of the total DTUD was scored with ‘Yes’ or ‘No’, and was considered acceptable if ≥90% of the informants evaluated the content of the DTUD as clear. Inter-rater reliability was assessed in a random subsample of 54 patients using pairs of independent ratings made by two clinicians present at the same admission interview. Assessment of the criterion validity of the DTUD was conducted in four out of six participating psychiatric clinics. Since a reference standard for the determination of need for highly specialized MDD care was not available, the experts’ clinical judgement constituted the reference standard. At each clinic, two clinicians with extensive clinical experience in the treatment of depressive disorders, independently and blinded to the index score (i.e. DTUD), made a clinical judgment based on the patient’s medical record as to whether the patient was in need of highly specialized care (Yes/No). An independent researcher verified the consistency between the two clinical judgments, and discrepancies were resolved by a consensus meeting with the first and second clinician.

### Statistical analysis

All analyses were conducted using SPSS (Statistical Package for the Social Sciences) version 20.0 (IBM SPSS Version 20, IBM, New York, NY, USA). Statistical significance was inferred at *P* < 0.05 (two-tailed). Demographic characteristics and feasibility outcomes were examined using descriptive statistics. Feasibility outcomes were evaluated according to the criteria outlined in the procedures section. Inter-rater reliability was assessed by Krippendorff’s alpha for individual items and total DTUD score [22, 23]. Krippendorff’s alpha is a conservative reliability estimate for judgments made by any number of raters, and is adaptable to any level of measurement [24]. For each of the estimated Krippendorff’s alpha values, 95% confidence intervals (CIs) were computed based on 10,000 bootstrap replications. Estimated Krippendorff’s alpha values were evaluated against the minimum recommended reliability level of 0.667 [23]. Convergent validity was assessed by Spearman’s correlation coefficients between total DTUD scale scores and total MSM and DM-TRD scores. Correlations of 0.10–0.30, 0.30–0.49 and > 0.50 were considered as weak, moderate and strong, respectively [25]. The DTUD was hypothesized to have a positive correlation with the MSM and DM-TRD. A receiver-operating characteristic (ROC) curve was generated to assess the criterion validity of the DTUD. In order to determine the optimal cut-off score, a Youden index (J = (sensitivity_c_ + specificity_c_) -1) [26] was calculated for a range of cut-off scores. The cut-off score that corresponded to the highest Youden index was selected as the optimal cut-off score.

## Results

### Description of the study population

From November 2015 to April 2016, a total of 243 patients were studied. Table 2 summarizes the main demographic and clinical data of the patients. The mean age of the patients was 44.22 years (SD = 12.64) and 60.49% (*n* = 147) were female. The length of the index depressive episode was less than twelve months for 44.45%; one year to two years for 11.52%, and more than two years for 44.03% of the sample. Using DSM-IV specifiers, the majority of the patients were diagnosed with moderate (36.63%) or severe MDD without psychosis (34.98%). The mean total DTUD score was 3.70 (SD = 2.00). Mean total MSM and DM-TRD scores were 6.71 (2.42) and 11.30 (3.67), respectively.

**Table 2:** Demographic and clinical characteristics of study sample

	IRR sample^a^	Criterion validity sample^a^	Total sample
N	54	132	243
Age, years			
Mean (SD)	41.48 (12.15)	44.67 (11.89)	44.22 (12.64)
Range	23–66	22–69	18–78
Sex (n, %)			
Male	24 (44.44)	57 (43.18)	96 (39.51)
Female	30 (55.66)	75 (56.82)	147 (60.49)
Duration of current MDD episode (n, %)			
Acute (≤12 months)	27 (50.00)	56 (42.42)	108 (44.45)
Subacute (13–24 months)	7 (12.96)	13 (9.85)	28 (11.52)
Chronic (> 24 months)	20 (37.04)	63 (47.73)	107 (44.03)
Symptom severity of current MDD episode (n, %)			
Mild	14 (25.93)	24 (18.18)	48 (19.75)
Moderate	25 (46.30)	47 (35.61)	89 (36.63)
Severe without psychosis	11 (20.37)	49 (37.12)	85 (34.98)
Severe with psychosis	4 (7.41)	12 (9.09)	21 (8.64)
Total DTUD score			
Mean (SD)	3.85 (1.85)	3.65 (2.05)	3.70 (2.00)
Range	0.00–8.00	0.00–9.00	0.00–9.00
Total MSM score			
Mean (SD)	6.02 (2.16)	6.98 (2.42)	6.71 (2.42)
Range	3.00–13.00	3.00–13.00	3.00–13.00
Total DM-TRD score			
Mean (SD)	10.55 (3.13)	11.60 (3.97)	11.30 (3.67)
Range	6.00–23.50	3.00–23.50	3.00–23.50

^a^ Part of total sample

*IRR* Inter-Rater Reliability, *DTUD* Decision Tool Unipolar Depression, *MSM* Maudsley Staging Method, *DM-TRD* Dutch Measure for quantification of Treatment Resistance in Depression

### Feasibility

The mean administration time was 4.49 min (SD = 2.71), and the content of the total DTUD was in 94.65% of the evaluations judged as clear. Two out of 48 clinicians suggested the addition of a mid-point in the set of response options, such as “maybe” or “don’t know”. Three out of 48 clinicians expressed concern about the clarity of the items “social factors maintaining the depression” (item 9) and “severe psychosocial dysfunctioning” (item 10), and suggested the inclusion of examples and descriptions of both items to improve item clarity. Another suggestion included the addition of a statement according to which grade of diagnostic validity item 3 (comorbid personality disorder) should be determined - i.e. whether the item is met in case of a diagnosed personality disorder according to a structured interview such as the Structured Clinical Interview for DSM-IV (SCID) [19]), or also on the basis of a clinically suspected comorbid personality disorder, without administration of a formal structured interview.

### Reliability

Inter-rater reliability was determined for 54 participants. As demonstrated in Table 3, the Krippendorf’s alpha value of the total DTUD score was 0.82 (95% CI 0.76–0.87). The Krippendorff’s alpha values of the individual items of the DTUD varied between 0.69 (95% CI 0.52–0.83) for comorbid personality disorder and 0.91 (95% CI 0.77–1.00) for comorbid substance use disorder. No item was below the minimum recommended reliability level of 0.667 [23].

**Table 3:** Krippendorff’s alpha values of the DTUD (*n* = 54, 95% CIs generated by 10,000 bootstrap replications)

Item		Krippendorff’s alpha (95% confidence interval)
1	Severity	0.81 (0.69–0.92)
2	Course	0.82 (0.68–0.92)
3	Comorbid personality disorder	0.69 (0.52–0.83)
4	Comorbid substance use disorder	0.91 (0.77–1.00)
5	Other psychiatric comorbidity	0.78 (0.64–0.90)
6	Somatic comorbidity	0.84 (0.64–0.92)
7	Coping	0.85 (0.74–0.94)
8	Childhood trauma	0.82 (0.70–0.92)
9	Social factors	0.78 (0.64–0.90)
10	Psychosocial functioning	0.73 (0.58–0.85)
Total DTUD score		0.82 (0.76–0.87)

*DTUD* Decision Tool Unipolar Depression

### Validity

**Fig. 1:**
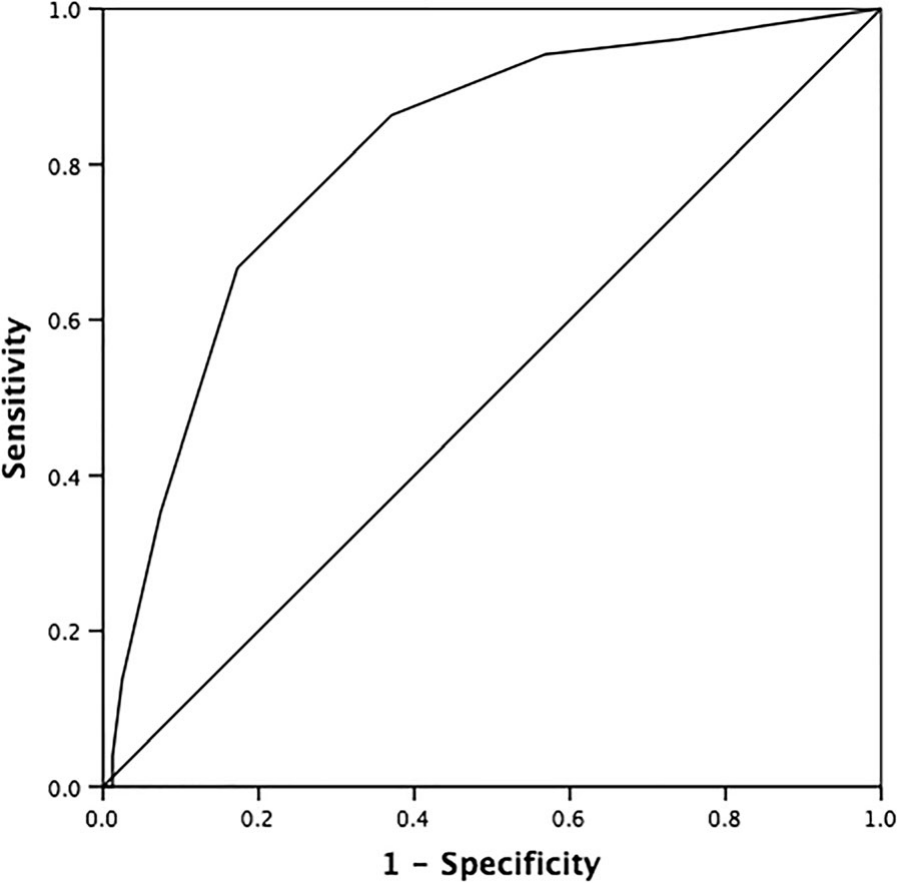
ROC curve for the DTUD (area under the curve (AUC) = 0.81)

As expected, higher scores on the DTUD were associated with higher scores on the MSM (r_s_ (241) = 0.47 *P* < 0.001) and DM-TRD (r_s_ (241) = 0.53, P < 0.001). Figure 1 and Table 4 summarize the operating characteristics of the DTUD. The area under the curve (AUC) was 0.81 (95% CI 0.73–0.87). Based on the maximum Youden index of 0.494, maximum discrimination was reached at a cut-off score of ≥5. This cut-off score demonstrated a sensitivity of 0.67 (95% CI 0.52–0.79) and a specificity of 0.83 (95% CI 0.73–0.90).

**Table 4:** Operating characteristics of the DTUD with the experts’ clinical judgment constituting the criterion standard

DTUD Scale Score	Sensitivity (95% CI)	Specificity (95% CI)	Youden index^a^
≥ 3	0.94 (0.84–0.99)	0.43 (0.32–0.55)	0.373
≥ 4	0.86 (0.74–0.94)	0.63 (0.52–0.73)	0.492
≥ 5	0.67 (0.52–0.79)	0.83 (0.73–0.90)	0.494
≥ 6	0.35 (0.22–0.50)	0.93 (0.85–0.97)	0.279

^a^ Youden index = (sensitivity + specificity) - 1

*DTUD* Decision Tool Unipolar Depression, *CI* Confidence Interval

## Discussion

This study evaluated the psychometric properties of the Decision Tool Unipolar Depression (DTUD) in the identification of patients with MDD in need of highly specialized mental healthcare. Overall, the results provide initial support for the psychometric properties of the DTUD. The DTUD demonstrated excellent feasibility and adequate inter-rater reliability. The associations with measures of TRD and health-related quality of life supported convergent validity. Furthermore, the DTUD demonstrated satisfactory criterion validity for use in clinical practice; a cut-off score of ≥5 was found to represent an optimal cut-off point for identifying patients with MDD in need of highly specialized care. The results support the use of the DTUD in busy, routine, outpatient specialized and highly specialized settings. Both the average completion time and content clarity of the questionnaire were within a-priori determined acceptability limits (≤10 min for completion time and ≥ 90% for clarity).

A noteworthy finding is that clinicians tend to disagree on the presence of a comorbid personality disorder. An analysis of the provided qualitative feedback regarding this item suggested that this may be due to the differential grade of diagnostic validity at which the presence of a comorbid personality disorder was determined (i.e. whether the item is met in case of a diagnosed personality disorder according to a structured interview, or also on the basis of a clinically suspected comorbid personality disorder, without the administration of a formal structured interview). Previous studies have shown that training on how to score an instrument improves the reliability of a scale [27, 28]. Whether training also improves the reliability of the DTUD should be studied in future research.

The pattern of correlations between the DTUD and measures of (future) TRD and health-related quality of life supported convergent validity. Specifically, the DTUD was more strongly associated with the DM-TRD than with the MSM, suggesting that the MSM measures a more distantly related concept. This is to be expected since the DM-TRD is an extension of the MSM, additionally including items for functional impairment, comorbid anxiety, personality disorders and psychosocial stressors [21], all of which are well-known factors associated with unfavourable treatment outcome in MDD [29–34]. In addition, the DTUD showed good discriminative validity relative to the experts’ clinical judgment of the need for highly specialized care (AUC = 0.81). Based on the Youden index, maximum discrimination was reached at a cut-off score of ≥5, with a sensitivity of 67% and a specificity of 83%. A lower cut-off point (≥4) produced a similar Youden index value with higher sensitivity (86%) but at the cost of a lower specificity (63%). Given the limited capacity and higher costs of highly specialized services [13], higher specificity should be prioritized in order to decrease the rate of false positives, hence, a score of ≥5 is recommend and should be tested in future Decision Tool guided studies. For patients obtaining a DTUD score of 4, an initial evidence-based treatment in specialized mental healthcare should be combined with systematic monitoring and in case of inadequate treatment response, a quick, prioritized referral to highly specialized care should be strongly considered.

The key strengths of this study are the broad age-range of the sample, the extensive set of psychometric properties studied, and the nation-wide representation of the participating clinical sites (six clinics from across the country), which adds to the generalizability of the results. Further, to our knowledge, this is the first study in which a selection algorithm is developed and validated that facilitates the early identification of patients with MDD in need of highly specialized care. The results should, however, also be viewed in light of some study limitations. First, the feasibility of the DTUD was evaluated by completion time and content clarity; future studies could also assess the feasibility of the DTUD with regard to item nonresponse. In the present study, an analysis of missing values was not possible since the web-based form was constructed in such a way that it required completion of all items. Second, the experts’ clinical judgement constituted the reference standard for the evaluation of the criterion validity, which may have introduced subjective error. However, in the absence of a gold standard test for the identification or patients with MDD in need of highly specialized care, the experts’ clinical judgement was considered the most adequate and clinically meaningful indication of highly specialized mental healthcare need. In addition, to reduce the subjective nature and increase the accuracy of the reference standard, the final clinical judgment was based on independent, dual examinations of comprehensive medical files by clinicians with extensive clinical experience in the treatment of depressive disorders. Third, the results reported in this paper represent a first examination of the DTUD psychometric properties. It was beyond the scope of this study to examine other issues, such as test-retest reliability, which should be examined in future studies. Fourth, it should be noted that the development of assessment tools typically requires a trade-off between feasibility (i.e. practicality) and validity (i.e. precision). Since the aim was to develop a simple, routine tool that is quick and easy to complete, the DTUD was constructed as a simple additive score of unweighted items. Future research might examine the relative importance of the individual items, as well the effect of the use of weighted items on the feasibility and validity of the DTUD. In addition, although the factors of the DTUD resulted as independent, distinct indicators of patients with MDD in need of highly specialized care from the concept mapping study [18], there might be a potential for reduction of DTUD items through merging of potentially correlated items. Since the evaluation of the effect of merging potentially correlated items on the psychometric properties of the DTUD would require a new operationalization of items and subsequent psychometric testing, this evaluation should be addressed in future studies. Moreover, although the currently recommended cut-off value will likely generalize to similar psychiatric settings in The Netherlands, this remains to be validated. Finally, since the financing and organization of mental healthcare systems varies internationally [35, 36], future studies are needed to determine the appropriate cut-off value for other countries. In this regard, adapting the DTUD into other languages to test its suitability in similar groups of patients but in different healthcare systems may be beneficial to extend its cross-national robustness.

## Conclusions

The results of the present study provide initial support for the psychometric properties of the DTUD. The DTUD proves to be a tool with good feasibility, reliability and validity and, therefore, is a promising instrument for the early identification of patients with MDD in need of highly specialized care. As such, the results of this study have the potential to facilitate the selection and initiation of the optimal initial treatment in patients with MDD, which in turn may improve the clinical effectiveness and cost-effectiveness of treatment strategies.

## Additional file

Additional file 1: English translation of the Decision Tool Unipolar Depression (DTUD). (PDF 177 kb)

## Data Availability

The datasets used and/or analysed during the current study are available from the corresponding author on reasonable request.

## Abbreviations

AUC: Area Under the Curve
CI: Confidence Interval
DM-TRD: Dutch Measure for quantification of Treatment Resistance in Depression
DSM: Diagnostic and Statistical Manual of Mental Disorders
DTUD: Decision Tool Unipolar Depression
IRR: Inter-Rater Reliability
MDD: Major Depressive Disorder
MSM: Maudsley Staging Method
ROC-curve: Receiver-Operating Characteristic curve
SCID-I: Structured Clinical Interview for DSM Axis I Disorders
TRD: Treatment Resistant Depression
