# Supplementary material for: The decision tool unipolar depression (DTUD): a new measure to facilitate the early identification of patients with major depressive disorder in need of highly specialized care
Source: BMC Psychiatry. 2019 Jun 11;19:179. doi: 10.1186/s12888-019-2165-9 (PMC6560755; doi:10.1186/s12888-019-2165-9)
Supplement: Supplementary file 1 — English translation of the Decision Tool Unipolar Depression (DTUD). (PDF 177 kb) [file 12888_2019_2165_MOESM1_ESM.pdf]

Decision Tool Unipolar Depression (DTUD) Version 1.0

Objective

The Decision Tool Unipolar Depression (DTUD) was developed to aid clinicians in the early identification of the subgroup of patients with MDD in need of highly specialized care. The DTUD is a 10-item clinician-administered instrument designed for use in routine psychiatric clinical practice to identify patients with MDD in need of highly specialized care during the diagnostic phase after referral. The total score is calculated by summing the scores of the ten items and ranges from 0 to 10.

The instrument is a tool for the assessment of highly specialised care need. It is possible to reasonably deviate from the outcome of the list, taking the patient's individual circumstances, patients' preferences, the individual's level of motivation and the available treatment resources.

Completing the decision tool will take up to 5 minutes.

Your findings are both relevant for discussion in a multidisciplinary consultation and in a consultation with the patient

NB The Dutch translation of the DTUD was tested for its psychometric properties; the psychometric properties of other translations are to be tested in future studies.

This work was funded by the Dutch Network for Quality Development in mental health care.

Decision Tool Unipolar Depression (DTUD) Version 1.0

Name of patient:

Patient medical record number:

1 Does the patient have severe depression?<br>Indication: HDRS ≥25; IDS-C/SR ≥39; Q-IDS-C/SR ≥16; MADRS ≥31; BDI ≥30; CGI serious; DSM-IV 296.x3; PHQ ≥20	◯ yes<br>◯ no	Findings and/or level
2 In the index episode, have there been any unsuccessful previous treatments in specialised mental health care **and** does the patient have a<br>- recurrent (more than 2 episodes in the past 5 years) **or**<br>- chronic (>2 years) course of depression?	◯ yes<br>◯ no	Findings and/or level
3 Does the patient have a comorbid personality disorder according to DSM-IV/5 criteria that interferes with the depression treatment?	◯ yes<br>◯ no	Findings and/or level
4 Does the patient have a comorbid substance dependence disorder that interferes with the depression treatment?	◯ yes<br>◯ no	Findings and/or level
5 Does the patient have other severe psychiatric comorbidity that interferes with the depression treatment?	◯ yes<br>◯ no	Findings and/or level
6 Does the patient have somatic comorbidity that interferes with the depression treatment?	◯ yes<br>◯ no	Findings and/or level
7 Does the patient have a disadaptive coping style that interferes with the depression treatment?<br>Hint: think of extreme avoidance or externalization	◯ yes<br>◯ no	Findings and/or level
8 Does the patient have a history of prolonged trauma/neglect in childhood?	◯ yes<br>◯ no	Findings and/or level
9 Are there any social factors contributing to the depression that are hard to influence?	◯ yes<br>◯ no	Findings and/or level
10 Does the patient exhibit severe psychosocial dysfunctioning?	◯ yes<br>◯ no	Findings and/or level

Total amount of positive (=yes) scores ≥5?

◯ yes ---> indicated for highly specialized care on the basis of the DTUD*

◯ no ---> not indicated for highly specialized care on the basis of the DTUD*

* This is a mental health care indication for adults. No rights can be derived.

© TOPGGz iMTA All rights reserved. This tool has been released for use and may be copied. It is not allowed to change the text.

For questions or for reporting experiences with the decision tool, please email us at info@topggz.nl
